# Magnesium intake and serum magnesium concentration in children with type 1 diabetes mellitus: association with glycemic control and clinical symptoms

**DOI:** 10.3389/fnut.2024.1477814

**Published:** 2024-12-20

**Authors:** Lesya Dobrovolska, Oksana Boyarchuk

**Affiliations:** Department of Children’s Diseases and Pediatric Surgery, I. Horbachevsky Ternopil National Medical University, Ternopil, Ukraine

**Keywords:** magnesium intake, hypomagnesemia, magnesium, type 1 diabetes mellitus, glycemic control, calcium, clinical signs

## Abstract

**Introduction:**

Magnesium is involved in numerous reactions that regulate the functioning of different organs and systems. Hypomagnesemia impacts on the development of various metabolic disorders, including insulin resistance and diabetes mellitus (DM). Studying magnesium levels in children with type 1 DM is crucial, as deficiencies are linked to many diabetes complications. The study aimed to determine dietary magnesium intake, serum magnesium concentration in children with type 1 DM, and their impact on the clinical course of DM.

**Methods:**

This case-control study involved 50 children with type 1 DM (cases) and 67 healthy children (control) aged 6–17 years. We conducted a survey to gather basic characteristics, weekly consumption of specific food items, and clinical data for patients with DM and healthy children. Additionally, serum magnesium, calcium, and phosphorus concentration were measured.

**Results:**

Insufficient magnesium dietary intake was observed in 46% of patients with DM and in 34.3% of healthy children (*p* > 0.05). Serum magnesium concentration in healthy children was higher than in children with DM (0.94 ± 0.24 vs. 0.84 ± 0.14, *p* = 0.011), although the proportion of children with hypomagnesemia did not differ between the groups (11.9% and 14.0%, respectively). Hypomagnesemia was more frequently observed in children from rural areas in both groups: 85.7% in children with DM (*p* = 0.054) and 62.5% in healthy children (*p* = 0.010). All children with hypomagnesemia had poor DM control compared to 61.3% of patients with normal magnesium concentration (*p* = 0.047). The mean magnesium concentration in children with optimal glycemic control was significantly higher than in children with poor control (0.96 ± 0.09 vs. 0.78 ± 0.14 mmol/L, *p* = 0.001). In DM children and hypomagnesemia, significant decreases in serum calcium and phosphorus concentrations were observed (*p* = 0.008 and *p* = 0.017, respectively). Headache and attention disorders were significantly more frequent in DM patients with hypomagnesemia (71.4% vs. 20.9%, *p* = 0.006; 28.6% vs. 4.7%, *p* = 0.031, respectively).

**Conclusion:**

The study demonstrates lower serum magnesium levels in children with type 1 DM than in healthy children, with a higher prevalence of hypomagnesemia in rural areas and those with poor glycemic control. Hypomagnesemia in DM children was associated with lower serum calcium and phosphorus levels, as well as more frequent symptoms such as headaches and attention deficits. Monitoring of serum magnesium is essential in routine care of children with DM.

## Introduction

Magnesium plays a crucial role in the body’s functions. It is involved in over 600 enzymatic reactions that regulate the functioning of the heart, blood vessels, neurons, muscles, and other organs and systems (1). Most of the magnesium is found in bones and soft tissues, with only 1% in the blood (2). Therefore, serum magnesium levels correlate poorly with total body magnesium levels or concentrations in specific tissues (3). Serum magnesium concentrations slightly depend on a child’s age and range from 0.70 to 0.95 mmol/L in children older than 5 months (4, 5), and serum levels below 0.7 mmol/L are defined as hypomagnesemia (2).

Symptoms of magnesium deficiency are non-specific and may mask signs of other nutrient deficiencies or non-specific symptoms of chronic diseases (6). Common causes of magnesium deficiency include insufficient dietary intake, impaired absorption in the gastrointestinal tract, kidney dysfunction, medications (diuretics, calcineurin inhibitors, and certain antibiotics), and genetic factors (2). Insufficient dietary intake is one of the most common factors of hypomagnesemia in children. Recommended magnesium intake varies by age and sex (7) and ranges from 75 mg in children aged 7–12 months to 410 mg in boys and 360 mg in girls aged 14–18 years (8). Several studies have shown insufficient dietary magnesium intake in adult patients in Europe and North America (7, 9). Data on magnesium intake from food in the pediatric population are limited, though insufficient dietary intake is noted, particularly in adolescents (10).

Numerous studies have demonstrated the impact of hypomagnesemia on the development of various metabolic disorders, including insulin resistance and diabetes mellitus (DM) (11). The frequency of hypomagnesemia ranges from 13.5% to 47.7% in patients with type 2 DM (12). On the other hand, high magnesium intake has been shown to prevent chronic metabolic complications (11). The positive effects of magnesium in diabetes include improved glucose and insulin metabolism, reduced chronic low-grade inflammation, protection of cells from oxidative stress and damage, improved lipid profile, enhanced endothelium-dependent vasodilation, and neuropathy prevention (2, 11, 13).

The aim of our study was to determine dietary magnesium intake, serum magnesium concentration in children with type 1 DM, and their impact on the clinical course of DM.

## Materials and methods

### Study design and participants

This case-control study included 50 children with type 1 DM (cases) and 67 healthy children (control) aged 6–17 years. The children with DM were examined during hospitalization in the endocrinology department of Ternopil regional children’s hospital, Ukraine. The control group children were examined during routine preventive check-ups at the outpatient department of city and regional children’s hospital in Ternopil, Ukraine. The study was conducted in the spring and autumn of 2021.

Inclusion criteria for the control group were the absence of chronic diseases, acute illnesses, and medication intake, along with informed consent from the children and/or their parents to participate in the study. Inclusion criteria for the DM group were a confirmed diagnosis of DM. Exclusion criteria for this group included the presence of other chronic diseases, kidney dysfunction, acute illnesses, and refusal of children and/or their parents to participate in the study.

Data collection involved a survey to gather basic characteristics (age, gender, place of residence, and parents’ education) and clinical data for patients with DM (complaints, medical history, medication, vitamin, mineral, and supplement intake).

In addition to the primary complaints related to DM, attention was paid to other symptoms that might indicate hypomagnesemia, such as headaches, dizziness, attention disorders, memory issues, depression, irritability, sleep disturbances, cramps, muscle weakness, tremors, and involuntary muscle spasms (14).

To assess dietary magnesium intake, a survey was conducted regarding the weekly consumption of specific food items. The list included major foods for children of different ages, especially those containing magnesium. Each child, under parental supervision, recreated their weekly diet by specifying the number of food portion for each food. Portion sizes were standardized (e.g., a cap or half a cup, teaspoon or tablespoon, slice, etc.). For younger children (ages 6–9), parents helped reconstruct the weekly diet.

Using a questionnaire based on a magnesium content database in food products (10), the average amount and sources of magnesium intake were determined. The total weekly magnesium intake and the average daily intake from food were calculated and compared with national and international recommendations for daily nutrient requirements in children (8, 10, 15).

Children with DM had certain dietary restrictions, such as a limited intake of baked goods, barley, millet, pearl barley, oats, legumes, and sour cream. Rice, semolina, pasta, salty cheeses, sweet curds, cream, fatty meats and fish, canned foods in oil, and caviar were excluded or significantly limited.

All children underwent comprehensive clinical examinations, including anthropometric measurements [weight, height, and body mass index (BMI)]. The level of glycemic control was determined based on glycated hemoglobin (HbA1c) levels. According to the ISPAD Clinical Practice Consensus Guidelines 2022 (16), HbA1c levels below 7% were considered optimal glycemic control, while levels above 7% indicated poor glycemic control.

Additionally, serum magnesium, calcium, and phosphorus concentration were measured. Blood samples were taken via venipuncture from the elbow vein using disposable “Vacutainer” systems on an empty stomach. Quantitative determination of magnesium, calcium, and phosphorus was performed using ELISA kits from Assay Kit Elabscience, USA, by a colorimetric method. All measurements were conducted in the same laboratory for all participants.

Written informed consent was obtained from all study participants or their parents before blood collection. The experimental protocol was conducted in accordance with the guidelines of the 1975 Declaration of Helsinki, revised in 2000, and approved by the I. Horbachevsky Ternopil National Medical University Ethics Committee (Minutes No 60 from 1 September 2020).

### Statistical analysis

Statistical analysis was conducted using the STATISTICA 10.0 statistical package and Microsoft Excel 2003. For normally distributed samples, mean values (*m*) and standard deviation (SD) were calculated. The data were processed using variation statistics methods. Student’s *t*-test was used to compare mean values. For non-normally distributed samples, data were presented as medians and interquartile ranges (IQR) [25%–75%]. Mann–Whitney *U*-test was used to compare indicators in two independent groups. Frequency indicators in the observation groups were compared using the χ^2^ test and Yates’ corrected χ^2^ test. Odds ratios (ORs) and 95% confidence intervals were determined to explore the influence of potential risk factors. Only statistically significant features were used for this analysis. Correlation analysis was performed by calculating Spearman’s rank correlation coefficient. Differences were considered significant at *p* < 0.05.

## Results

### Characteristics of DM patients and healthy children

Baseline characteristics of observed children with type I DM (cases) and healthy children (control) are presented in Table 1. Boys predominated among children with DM (62%), while there was no significant gender difference among healthy children. There was no significant difference in place of residence among patients with DM, and most parents (80%) had secondary education (*p* < 0.0001). In the group of healthy children, urban residents predominated with high significance, and higher education was observed in 52.2% of parents. There was no significant difference in age and BMI between the groups of children with DM and healthy children. Calcium and phosphorus levels did not differ between the groups. The average duration of DM in children was 4.95 ± 4.38 years, ranging from 1 week to 14 years. The average HbA1c level in children with type I DM was 8.83 ± 2.77%, ranging from 5.5% to 15.8%. Optimal glycemic control was observed in 31.6% of patients, while poor control was noted in 68.4%, among which 10 (26.3%) patients had newly diagnosed diabetes.

**TABLE 1:** Baseline characteristics of observed patients with type I diabetes mellitus (cases) and healthy children (control).

Characteristic	DM patients, *n* = 50	Healthy children, *n* = 67
Male, *n* (%)	31 (62.0)	35 (52.2)
Female, *n* (%)	19 (38.0)	32 (47.8)
Place of residence		
Rural, *n* (%)	26 (52.0)	17 (25.4)*
Urban, *n* (%)	24 (48.0)	50 (74.6)
Parents’ education		
Higher, *n* (%)	8/40 (20.0)	35/67 (52.2)*
Secondary, *n* (%)	32/40 (80.0)	32/67 (47.8)
Age at visit, years (mean ± SD)	12.29 ± 3.60	11.69 ± 3.67
DM duration, years (mean ± SD, min–max)	4.95 ± 4.38 (1 week–14 years)	–
BMI, kg/m^2^ (mean ± SD, min–max)	18.05 ± 3.33 (13.18–25.33)	19.65 ± 4.09(13.17–28.26)
HbA1c, % (mean ± SD, min–max)	8.83 ± 2.77 (5.50–15.8)	–
Glycemic control		
Optimal (HbA1c < 7%), *n* (%)	12/38 (31.6)	
Poor (HbA1c > 7%), *n* (%)	26/38 (68.4)	
Calcium, mmol/L (mean ± SD)	2.04 ± 0.26	2.08 ± 0.18
Phosphorus, mmol/L (mean ± SD)	1.19 ± 0.30	1.08 ± 0.27

*p < 0.01.

**FIGURE 1:**
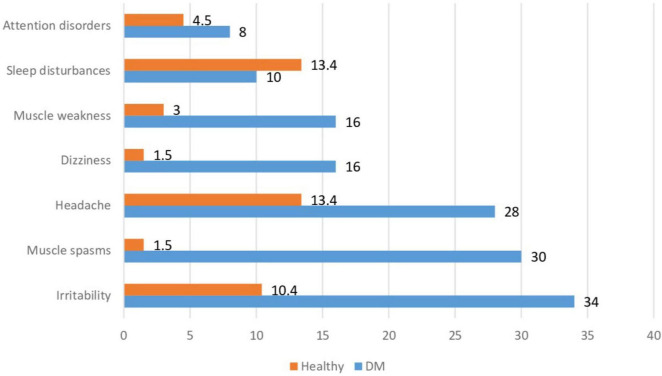
Frequency of non-specific symptoms in patients with type 1 DM and healthy children.

Specific symptoms of DM, such as polyuria and polydipsia, were present in 11 (22.0%) children at the time of the examination, mostly in those with newly diagnosed or poorly controlled DM. The frequency of non-specific symptoms in children with DM and healthy children is shown in Figure 1. Among the non-specific complaints in children with DM, irritability (34%), muscle spasms (30%), headache (28%), dizziness (16%), and muscle weakness (16%) were most commonly reported.

Healthy children significantly less often reported these non-specific symptoms, with headache being the most common – 9 (13.4%), sleep disturbances – 9 (13.4%), and irritability – 7 (10.4%). Children with DM more frequently reported irritability, muscle spasms, headache, dizziness, and muscle weakness compared to healthy children (*p* = 0.002; *p* < 0.001; *p* = 0.005; *p* < 0.001; *p* = 0.013, respectively).

### Dietary magnesium intake and serum magnesium concentration

The daily dietary magnesium intake and serum concentrations in patients with type 1 DM and healthy children is shown in Table 2. The median values of dietary magnesium intake did not differ between the group of children with DM and healthy children. The percentage of children with DM whose magnesium intake was below the recommended age norms was 1.34 times higher than the corresponding percentage of healthy children, although the difference was not statistically significant (*p* = 0.201).

**TABLE 2:** Daily dietary magnesium intake and serum magnesium concentration in DM and healthy patients.

Patients’ group	*n*	Daily dietary intake, median [interquartile range]	Insufficient dietary intake, *n* (%)	Serum magnesium, mmol/L (mean ± SD)	Hypomagnesemia, *n* (%)
DM patients	50	270.83[210.62–367.19]	23 (46.0)	0.84 ± 0.14	7 (14.0%)
Healthy children	67	268.75[198.94–349.18]	23 (34.3)	0.94 ± 0.24	8 (11.9%)
*p*		0.939	0.201	0.011	0.742

It should be noted that in both groups (cases and control), insufficient dietary magnesium intake was more frequently observed in the 12–17 age group than in the 6–11 age group (4% vs. 42% in the DM group, *p* = 0.001, and 3% vs. 31.3%, in the healthy children, *p* < 0.0001).

**FIGURE 2:**
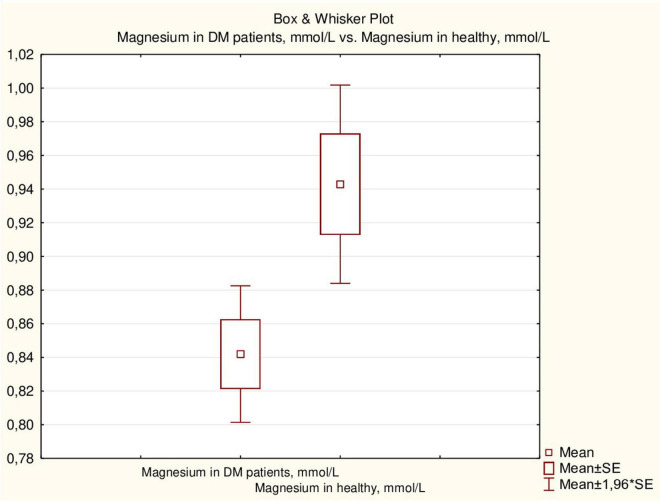
Serum magnesium concentrations in patients with type 1 DM and in healthy children.

Serum magnesium concentration in healthy children was higher than that in children with DM (*p* = 0.011) (Figure 2), although the proportion of children with hypomagnesemia did not differ between the two groups (14.0% and 11.9%, respectively).

### Magnesium status and its effects on glycemic control and clinical symptoms

Based on the serum magnesium concentration, children in both groups were divided into two subgroups: those with normal magnesium concentration and those with hypomagnesemia (Table 3). Baseline characteristics and clinical indicators were determined according to the magnesium concentration in patients with DM and in healthy children. No effect of gender on magnesium status was found in either group. However, hypomagnesemia was more frequently observed in children from rural areas in both groups: 85.7% in children with DM and 62.5% in healthy children (*p* = 0.054 and *p* = 0.010, respectively). Living in a rural area influence on hypomagnesemia (OR – 6.0156; 95% CI: 1.7788–20.3440; *p* = 0.004).

**TABLE 3:** Basic and clinical characteristics of patients with DM and healthy children depends on magnesium status.

Characteristic	DM patients, *n* (%)		*p*	Healthy children, *n* (%)		*p*
	**Normal magnesium**	**Hypomagnesemia**		**Normal magnesium**	**Hypomagnesemia**	
	***n* = 43**	***n* = 7**		***n* = 59**	***n* = 8**	
Male, *n* (%)	28 (65.1)	3 (42.9)	0.261	33 (55.9)	2 (25.0)	0.100
Female, *n* (%)	15 (34.9)	4 (57.1)	0.261	26 (44.1)	6 (75.0)	0.100
Place of residence			0.054			0.010*
Rural, *n* (%)	20 (58.1)	6 (85.7)		12 (20.3)	5 (62.5)	
Urban, *n* (%)	23 (41.9)	1 (14.3)		47 (79.7)	3 (37.5)	
Parents’ education level			0.145			0.536
Higher, *n* (%)	8 (24.2)	0 (0)		30 (50.8)	5 (62.5)	
Secondary, *n* (%)	25 (75.8)	7 (100)		29 (49.2)	3 (37.5)	
Age at visit, years (mean ± SD)	12.27 ± 3.60	12.43 ± 3.37	0.913	11.50 ± 3.46	14.50 ± 3.20	0.027*
BMI, kg/m^2^ (mean ± SD)	17.75 ± 3.23	19.86 ± 3.94	0.126	19.19 ± 4.12	22.56 ± 2.97	0.031*
BMI, percentile	41.04 ± 31.83	55.47 ± 40.84	0.289	60.33 ± 30.48	74.21 ± 22.87	0.224
DM duration, years (mean ± SD)	5.02 ± 4.34	4.50 ± 4.59	0.777			
HbA1c, % (mean ± SD)	8.63 ± 2.84	10.08 ± 1.53	0.313			
Glycemic control, *n* (%)	*n* = 31		0.047*			
Optimal	12 (38.7)	0 (0)				
Poor	19 (61.3)	7 (100)				
Magnesium, mmol/L (mean ± SD)	0.87 ± 0.13	0.65 ± 0.04	<0.001**	0.99 ± 0.21	0.60 ± 0.09	<0.001**
Daily dietary intake, median [IQR]	296.88 [210.62–377.21]	221.71 [217.66–303.37]	0.131	268.75 [202.55–328.90]	257.98 [105.23–492.05]	0.779
Calcium, mmol/L (mean ± SD)	2.09 ± 0.23	1.78 ± 0.26	0.008*	2.09 ± 0.15	1.99 ± 0.25	0.139
Phosphorus, mmol/L (mean ± SD)	1.24 ± 0.27	0.89 ± 0.39	0.017*	1.12 ± 0.26	0.86 ± 0.15	0.061
Frequency of non-specific symptoms, *n* (%)	27 (62.8)	6 (85.7)	0.236	20 (33.9)	3 (37.5)	0.840
Irritability, *n* (%)	15 (34.9)	2 (28.6)	0.744	7 (11.9)	0 (0)	0.303
Muscle spasms, *n* (%)	12 (27.9)	3 (42.9)	0.423	1 (1.7)	0 (0)	0.711
Headache, *n* (%)	9 (20.9)	5 (71.4)	0.006*	8 (13.6)	1 (1.3)	0.934
Dizziness, *n* (%)	7 (16.3)	1 (14.3)	0.894	1 (1.7)	0 (0)	0.711
Muscle weakness, *n* (%)	7 (16.3)	1 (14.3)	0.894	2 (3.4)	0 (0)	0.597
Sleep disturbances, *n* (%)	5 (11.6)	0 (0)	0.342	7 (11.9)	2 (2.5)	0.307
Attention disorders, *n* (%)	2 (4.7)	2 (28.6)	0.031*	3 (5.1)	0 (0)	0.514

Statistically significant values: *p < 0.05, **p < 0.001.

Parental education did not affect the magnesium status in either group. In the group of patients with DM, the mean age of the children did not differ depending on the magnesium status, but the mean age of healthy patients with hypomagnesemia was higher than that of children with normal magnesium levels (*p* = 0.027). Accordingly, similar trends were observed for BMI, which was higher in children with hypomagnesemia than in patients with normal serum magnesium concentration, but the difference was statistically significant only in the group of healthy children (*p* = 0.031). However, there was no difference in BMI percentiles between groups with hypomagnesemia and normal magnesium concentration in both groups.

**FIGURE 3:**
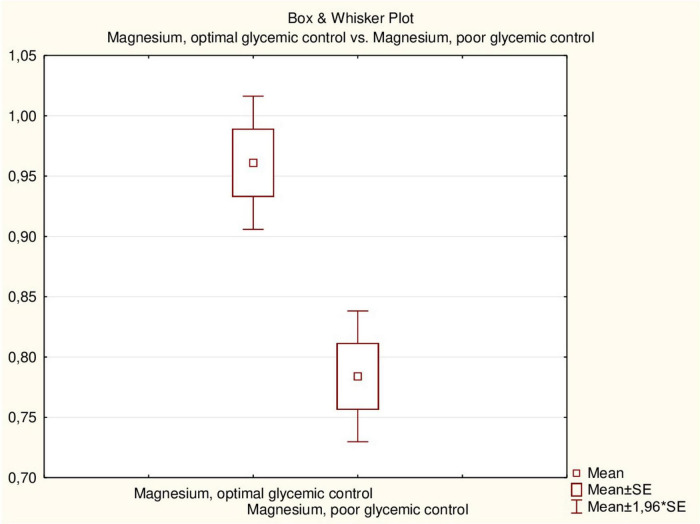
Serum magnesium concentration (mmol/L) in patients with type 1 DM depends on glycemic control.

**FIGURE 4:**
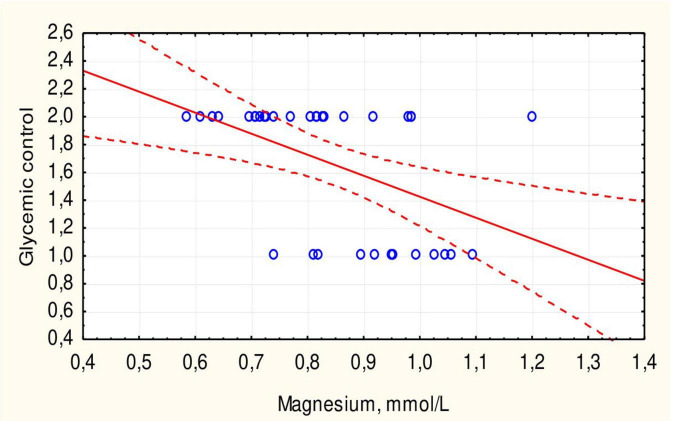
Correlation between serum magnesium concentration and glycemic control in children with type 1 DM.

The mean duration of DM did not differ between children with hypomagnesemia and those with normal magnesium concentration. The mean HbA1c level was somewhat higher in patients with hypomagnesemia, but the difference was not statistically significant (*p* = 0.313). There was no significant correlation between HbA1c levels and magnesium concentration in children with DM (*r* = 0.3251, *p* > 0.05). However, all children with hypomagnesemia had poor DM control compared to 61.3% of patients with normal magnesium concentration (*p* = 0.047). The mean magnesium concentration in children with optimal glycemic control was significantly higher than in children with poor control (0.96 ± 0.09 vs. 0.78 ± 0.14 mmol/L, *p* = 0.001) (Figure 3). Additionally, there was an inverse correlation between serum magnesium levels and glycemic control (Figure 4).

The median value of daily magnesium intake in children with DM was higher in those with normal blood magnesium concentration, but the difference was not statistically significant (*p* = 0.131). In children with DM and hypomagnesemia, significant decreases in serum calcium and phosphorus concentrations were observed (*p* = 0.008 and *p* = 0.017, respectively). In healthy children, changes in phosphorus and calcium levels due to hypomagnesemia were not significant (*p* > 0.05).

Comparing the frequency of non-specific symptoms in children with DM depending on magnesium status, found that headache and attention disorders were significantly more frequent in patients with hypomagnesemia (71.4% vs. 20.9%, *p* = 0.006; 28.6% vs. 4.7%, *p* = 0.031, respectively). Additionally, OR was determined for significant indicators. Hypomagnesemia was found to influence the occurrence of headache [OR – 9.4444; 95% CI (1.5660–56.9603); *p* = 0.014] and attention disorders [OR – 8.2000; 95% CI (0.9374–71.7305); *p* = 0.057]. In the group of healthy children, no difference in the frequency of symptoms was observed between children with normal magnesium concentrations and those with hypomagnesemia.

## Discussion

This study aimed to assess dietary magnesium intake and serum magnesium concentration in children with DM compared to healthy peers aged 6–17 years, focusing on the implications of hypomagnesemia on glycemic control and its relationship with calcium and phosphorus levels.

No significant difference was found in dietary magnesium intake between children with DM and healthy children. Studies on magnesium intake are quite limited, especially in children. According to the National Health and Nutrition Examination Survey (NHANES) for 2013–2016, 48% of Americans of various ages consume less magnesium from food and beverages than needed (10). The study also showed low magnesium dietary intake in adolescents. Another study indicated that 66% of adult non-users of dietary supplements had inadequate mineral intakes (17). Our study also revealed more frequent inadequate magnesium dietary intake in adolescents, both healthy and with DM (70% and 61.8%, respectively). The inadequate magnesium intake observed, especially in adolescents, is concerning given the increased dietary needs during this growth phase (8). Previous studies on dietary magnesium intake in patients with DM mainly focused on adults with type 2 DM (16, 17). Overall, 23.5% of patients with type 2 DM had inadequate magnesium intake (18). A meta-analysis demonstrated an inverse association between magnesium intake and the risk of type 2 diabetes (19).

The lower serum magnesium concentration in children with DM compared to healthy children is clinically significant (*p* = 0.011), suggesting that magnesium deficiency may contribute to complications associated with diabetes (20). Researchers suggest that there may be an association between impaired antioxidant protection and magnesium deficiency in children with type 1 DM (21, 22). The frequency of hypomagnesemia in children with DM was 14% and did not significantly differ from that in healthy patients (*p* > 0.05). Other studies reported a hypomagnesemia frequency of 3.4% in children with type 1 DM, also not significantly different from healthy children (23). Some researchers indicate that about 10% of hospitalized patients have magnesium deficiency (5).

Hypomagnesemia was more frequently observed in rural residents, both in healthy children and in patients with DM, probably due to potential dietary access differences. The OR indicated that living in rural areas may be a risk factor for hypomagnesemia. Separate studies have shown the impact of low magnesium and potassium intake in rural areas on the development of type 2 DM (24).

Hypomagnesemia was more common in children with DM with poor glycemic control, as demonstrated in other studies (13, 22, 25, 26). Less than a third of patients had optimal glycemic control, while the rest had poor control, consistent with the results of our previous study with a larger number of patients (27, 28). The average serum magnesium concentration in children with optimal glycemic control was significantly higher than in children with poor control (*p* = 0.001). A negative correlation between serum magnesium levels and glycemic control was also established (Figure 3). This negative correlation (*r* = 0.4712, *p* < 0.05) indicates that hypomagnesemia may exacerbate glycemic dysregulation in children with DM. Other researchers suggest that hypomagnesemia in adult DM patients is due to insulin resistance, a sign of type 2 diabetes (29). The authors also did not note a correlation between HbA1c levels and magnesium concentration, which was also demonstrated in our study. Similar trends of hypomagnesemia affecting glycemic control were noted in adults with type 2 DM (18, 30). However, another study showed a negative correlation with HbA1c % in children with type 1 DM (22).

In children with DM, hypomagnesemia affected serum calcium and phosphorus levels. Lower serum calcium and phosphorus levels in children with DM and hypomagnesemia (*p* = 0.008 and *p* = 0.017, respectively) highlight the potential risk for compromised bone health in this population. Such changes were not observed in healthy children. Another study showed that serum magnesium concentration positively correlated with calcium and phosphorus levels (13). Magnesium is involved in the transport of potassium and calcium ions and maintains their levels in the blood (1). Electrolyte imbalance due to hypomagnesemia was most pronounced in patients with DM. Hypomagnesemia results in decreased levels of parathyroid hormone and vitamin D3, which can affect calcium-phosphorus metabolism and impair bone resorption (31). Magnesium influences bone cell growth and formation and its strength (15). The role of hypomagnesemia in the development of osteoporosis is also well-established (32).

The symptoms of hypomagnesemia are not specific and may be associated with the underlying disease and other deficiency states, including hypocalcemia (14, 33). While muscle cramps and headaches are common symptoms, their persistence in children with DM may indicate underlying metabolic disturbances that could impact overall health and quality of life (32). We collected symptoms that may be associated with hypomagnesemia (Figure 1 and Table 3). In children with DM and hypomagnesemia, headaches, and attention disorders were more common. Although these symptoms are multifactorial, the OR indicated that hypomagnesemia in children with DM could contribute to headaches and tended to affect attention disorder symptoms. These patterns were observed only in children with DM. Overall, it is suggested that symptomatic magnesium deficiency due to low dietary intake in healthy individuals is rare since the kidneys limit the excretion of the mineral in case of its deficiency (32). However, insulin resistance and/or type 2 diabetes increase magnesium excretion in the urine. Magnesium loss is considered a secondary cause of poor glycemic control and high glucose concentrations in the kidneys, which increase urine output (15). Nonetheless, other studies showed that increased magnesium intake reduced the risk of developing DM (19) and improved glycemic control in DM patients (34, 35).

Clinical signs and symptoms of hypomagnesemia are thought to appear at serum magnesium levels below 0.5 mmol/L, although this level was not observed in any of our children.

### Strengths and limitations

Research on magnesium dietary intake in DM patients was conducted for the first time among the Ukrainian pediatric population. The control group of children of different ages allowed for comparison with healthy children, strengthening the study. We determined the contribution of hypomagnesemia to certain symptoms observed in children with DM and other conditions.

While this study provides valuable insights into magnesium intake among the Ukrainian pediatric population, the small sample size and single-center design limit the generalizability of our findings. However, the study allowed us to identify certain patterns. Conducting a multicenter study involving more patients and more indicators will help identify other effects of hypomagnesemia on the course of DM in children.

## Conclusion

Mean serum magnesium concentration in patients with type 1 DM was lower than in healthy children, although there was no difference in dairy magnesium intake. Hypomagnesemia was more frequently observed in rural children, both those with type1 DM and healthy ones and was associated with poor glycemic control in children with DM. Additionally, children with type 1 DM and hypomagnesemia had lower serum calcium and phosphorus levels and more frequent symptoms such as headaches and attention deficits. These findings underscore the need for routine screening of magnesium levels in children with DM, particularly those in rural areas, to prevent potential complications associated with hypomagnesemia. Further research is needed to explore the other impact of hypomagnesemia on the clinical course of DM in children.

## Data Availability

The raw data supporting the conclusions of this article will be made available by the authors, without undue reservation.
